# Load and Recovery Monitoring in Top‐Level Youth Soccer Players: Exploring the Associations of a Web Application‐Based Score With Recognized Load Measures

**DOI:** 10.1002/ejsc.70031

**Published:** 2025-08-06

**Authors:** Jan Anderegg, Stefanie L. Brefin, Claudio R. Nigg, David Koschnick, Claudia Paul, Sascha Ketelhut

**Affiliations:** ^1^ School of Health Professions Institute of Physiotherapy Zurich University of Applied Sciences Winterthur Switzerland; ^2^ Department of Health Science Institute of Sport Science University of Bern Bern Switzerland; ^3^ Department of Education and Psychology Division of Health Psychology Freie Universität Berlin Berlin Germany; ^4^ Commbuddy UG Berlin Germany

**Keywords:** injury risk reduction, load monitoring, performance optimization, recovery monitoring, youth soccer

## Abstract

This study investigates the relationship between a web application‐based load and recovery score (LRS) and established load parameters. Seventy‐eight elite youth soccer players were recruited from a single top‐tier Swiss club. All participants were healthy and injury‐free at baseline and actively competing at the highest national youth level, participating in five training sessions per week. Players with recent injuries or chronic health conditions were excluded. Seventy‐one players (32.4% female) with an average age of 18 years (SD = 1.2) met the inclusion criteria and were monitored throughout ≥ 35 days, applying a repeated‐measure design. Daily assessments of the self‐reported LRS, along with measurements of player and trainer session ratings of perceived exertion, total distance covered, and total distance > 20 km/h, were collected. Linear mixed‐effects models were used to analyze the influence of load parameters on the following day's LRS. All training and match load parameters demonstrated significant negative correlations with the subsequent day's LRS. Player and trainer session ratings of perceived exertion had similar fixed effects (−0.013, 95% CI [−0.017, −0.010] vs. −0.008, 95% CI [−0.011, −0.006]), whereas total distance covered exhibited stronger associations (−0.668, 95% CI [−0.979, −0.355]) than total distance > 20 km/h (−0.009, 95% CI [−0.012, −0.006]). The impact of the different load parameters varied across groups and individuals. The LRS provides an easy‐to‐use digital tool that summarizes multiple training and recovery factors into one score, helping coaches and staff monitor player readiness in daily field settings. By offering accessible daily feedback, the LRS may help tailor training loads, manage recovery, and reduce the risk of overtraining and injuries.

## Highlights

- This study introduces the load and recovery score (LRS), a web application‐based tool combining subjective and objective load metrics to monitor athlete recovery and performance.
- The LRS shows significant negative associations with training and match load measures when controlled for repeated within‐subject measurements. This means that higher training and match loads are consistently reflected in lower LRS scores on the following day, highlighting the scale's capacity to capture the fatigue and recovery state of players.
- The LRS simplifies recovery monitoring by consolidating multiple metrics into a single score, helping coaches adjust training loads and reduce the risk of overtraining and injuries.

## 1 Introduction

Systematic assessment of load and recovery in athletes is essential for effectively adjusting training demands and implementing appropriate recovery strategies (Kellmann et al. 2018; Ketelhut and Ketelhut 2020). These adjustments can support athletes, coaching staff, and medical teams in preventing nonfunctional overreaching, overtraining, and the risk of subsequent injuries and illnesses, while also promoting performance optimization (Bourdon et al. 2017; Kellmann et al. 2018; Taylor et al. 2012). Various physiological, psychological, and biochemical markers are available to monitor training and match load, fatigue, and recovery (Bourdon et al. 2017). However, no single valid measurement tool exists that can serve this purpose comprehensively (Taylor et al. 2012). Some markers are impractical for field use due to their invasive nature (e.g., blood lactate concentration) or the excessive fatigue they induce (e.g., maximal power tests). Additionally, many stand‐alone monitoring procedures have been criticized (Lolli et al. 2019; Petrigna et al. 2019). Despite their high practicality in sports settings and strong correlation with training load, self‐reported assessments are also subject to criticism, as athletes may adjust their responses based on social desirability (Saw et al. 2016). Therefore, to encourage an unbiased response behavior, clear and open communication of guidelines regarding data access, their utilization, and the benefits resulting from monitoring is essential (Saw et al. 2017). Given these considerations, expert consensus in the field of load and recovery monitoring emphasizes the importance of adopting a multivariate approach to assess both load and recovery (Bourdon et al. 2017; Kellmann et al. 2018; Heidari et al. 2019). However, especially in team sports, such assessments must be conducted quickly, noninvasively, and with minimal additional burden on the athletes (Thorpe et al. 2017).

Although various tools exist, there remains a lack of practical, user‐friendly instruments that integrate both load and recovery information into a single, actionable score suitable for daily use in field settings. Existing measures often focus only on single aspects of fatigue or recovery (Bourdon et al. 2017). This limits their effectiveness for daily decision‐making by coaches and practitioners (Heidari et al. 2019). Furthermore, limited research has examined whether a combined score that integrates both subjective and objective load indicators can reliably reflect day‐to‐day fluctuations in an athlete's recovery status in real‐world, high‐performance team sport environments (Brefin et al. 2025).

To address these needs, a new web application‐based load and recovery score (LRS) with eight distinct subscales was developed to support competitive sports. The items within these subscales assess athletes' recovery status as well as their training and match loads. These parameters are combined into an overall score intended to assist athletes, coaches, and medical staff in adjusting training and competition loads and in tailoring recovery interventions based on the athlete's current condition. This approach aims to prevent overtraining, reduce injury risk, and optimize performance. This study aims to determine whether the LRS accurately reflects training and match load in a real‐world, high‐performance environment. The validation of the LRS in this study is operationalized by examining its associations with well‐established training and match load indicators. Training and match load were quantified through subjective measures—player session rating of perceived exertion (PSRPE) and trainer session rating of perceived exertion (TSRPE)—as well as objective measures, including total distance covered (TD) and total distance above 20 km/h (TD20). These parameters are widely used in elite sports to monitor internal and external load and have demonstrated sensitivity to fatigue and recovery in prior research (Bourdon et al. 2017; Brink et al. 2010; Haddad et al. 2017; Inoue et al. 2022; Jones et al. 2017; Rowell et al. 2017; Saw et al. 2016). Using a repeated‐measures longitudinal design over 5–7 weeks, this study tracked elite youth players daily to evaluate how fluctuations in these load measures predict next‐day LRS scores, controlling for intraindividual variability. A valid tool would show consistent negative associations between the previous day's training/match load and the next day's LRS, indicating sensitivity to load‐induced changes in recovery. Through this methodology, this study aims to provide empirical evidence that the LRS can serve as a practical, multifactorial metric reflecting load and recovery dynamics in a high‐performance sports environment.

## 2 Materials and Methods

### 2.1 Participants

Seventy‐eight male and female athletes from the U18, U19, and U21 teams of the Swiss soccer club were recruited for this study. All players were actively involved in these high‐performance teams, which play in the highest national youth leagues in Switzerland. Recruitment was facilitated by the junior division management and team coaches, who informed players about the study objectives during preseason information sessions. Participation was voluntary, and no incentives were provided.

Because no prior studies with the same objective exist to provide a statistical basis for estimating the required sample size, a formal power analysis could not be conducted. Consequently, a convenience sampling approach was applied based on team availability and player eligibility. Inclusion criteria were as follows: active membership in one of the specified teams, regular participation in team training, possession of a smartphone with internet access for daily data entry, and no long‐term illnesses or injuries at the time of data collection. Players who were injured or medically restricted at baseline were excluded from participation.

This study was conducted according to the World Medical Association's Declaration of Helsinki. All participants gave informed consent. For participants under the age of 18, informed consent from their legal guardian was obtained. This study was approved on the fifth of July, 2022, by the faculty of humanities ethics committee of the (blinded for review).

### 2.2 Study Design

A repeated‐measures design was employed using a 5‐ to 7‐week prospective longitudinal data collection period. This design allowed each athlete to serve as their own control over time, improving sensitivity to within‐subject changes in load and recovery. During this time, the players participated in their regular team training, which consisted of four team training sessions and one individual training session per week. The training sessions primarily focused on technical drills, tactical exercises, and athletic training. The athletic training emphasized endurance and preparation for the competitive season. The dependent variable (LRS) and four independent load variables (PSRPE, TSRPE, TD, and TD20) were repeatedly measured over time in the same athletes to capture short‐term within‐individual fluctuations in response to varying training and match loads. The repeated‐measures design enabled the modeling of both intraindividual variability and between‐subject differences, while accounting for clustering within players using linear mixed‐effects models.

The female U19 team was monitored for 35 consecutive days, starting on July 26, 2022. The male U18 and U21 teams were monitored for 52 consecutive days, beginning on July 27, 2022. During this period, players were asked to complete the web application‐based LRS on a daily basis. Both players and their respective coaches recorded training and match loads each day. GPS tracking and accelerometry data were collected on 26 occasions for the U18 team and 28 occasions for the U21 team. Because of organizational constraints, these measurements could only be collected on six days for the U19 team. Before the start of the measurement periods, two onboarding sessions were conducted with the players and their respective staff. These sessions involved account creation, instructions on using the web application, and detailed explanations of the measurement procedures. The junior division management, along with several coaches and athletic trainers, was involved in selecting feasible outcome measures and planning recruitment and instructions. Players were not involved in the study's design, recruitment, or conduct.

### 2.3 Measures

#### 2.3.1 Demographic Data

Demographic data were provided by the team's coaches and included team affiliation, playing position, age, height, body mass, and current occupation before the start of the monitoring period.

#### 2.3.2 Load and Recovery Score (LRS)

The players recorded values on eight distinct subscales daily through the web application. These subscales were carefully selected to capture both physical and psychological aspects of training load and recovery that are known to influence performance and risk of overtraining (Bourdon et al. 2017; Kellmann et al. 2018). Physical performance capability, overall recovery, muscular stress, and fatigue represent immediate physical readiness and signs of strain. Mood and sleep quality reflect mental well‐being and recovery quality, which can affect training response (Nyenhuis et al. 1997). HRV provides an objective measure of autonomic nervous system status (Buchheit 2014; Le Meur et al. 2013), whereas the ACWR accounts for recent training load spikes that may increase injury risk (Gabbett 2016). Recording these subscales daily allowed us to monitor short‐term fluctuations in each player's status and to generate an overall LRS that integrates these multiple dimensions.

Physical performance capability, overall recovery, and muscular stress were assessed by asking players to rate how much a set of adjectives related to each aspect applied to them at that moment (Hitzschke et al. 2015). Responses were given on a scale ranging from 0 (“does not apply at all”) to 6 (“fully applies”) for each of these three subscales (Hitzschke et al. 2015). Fatigue was evaluated using the question, “Do you feel exhausted?” (translated from German), with players answering on a Likert scale from 0 (“fully applies”) to 6 (“does not apply at all”). Mood was assessed using the question, “How do you feel today?” (translated from German), with responses ranging from 0 (“sad”) to 6 (“happy”). Sleep quality was rated by players, who assessed the quality of their previous night's sleep on a scale from 0 (“very bad”) to 6 (“very good”).

Players performed daily HRV measurements, recording their resting HRV upon waking using a heart rate monitor and chest strap (Polar H10, Polar Electro Oy, Kempele, Finland) along with the Elite HRV application (Elite HRV Inc., Asheville, United States) (Perrotta et al. 2017). The results were analyzed and interpreted according to established recommendations for the use of HRV in monitoring training status (Buchheit 2014; Le Meur et al. 2013; Plews et al. 2012). During the first week of the observation period, players tracked their root mean square of successive RR interval differences (RMSSD) values daily for 1 week, entering this data into the application to establish baseline values. In the following weeks, any RMSSD deviation from the baseline of more than 5% over three consecutive days led to a reduction of one point in the HRV‐Subscale‐Score. If the values remained below baseline for two additional days, the score was further reduced by one point. When RMSSD returned to baseline and stayed within the baseline range for two consecutive days, the subscale score increased by one point. If RMSSD remained within the baseline value for three consecutive days, the score was restored to 6.

To calculate the ACWR, training intensity was assessed after each session (using a scale from 0 to 10) and multiplied by the session's duration (in minutes). The total weekly value was then compared with the average of the previous 1–4 weeks (Gabbett 2016). A deviation greater than 1.5 resulted in a reduction of one point in the ACWR‐Subscale‐Score, whereas a deviation greater than 1.8 resulted in a reduction of two points. If the deviation was less than 1.5, the score returned to the baseline value of 6. Subsequently, the daily subscale scores were combined to generate the LRS (ranging from 0 to 120), with a higher LRS score indicating better recovery. The LRS is calculated as the sum of the subscales, weighted by factors of 3 (for physical performance capability, overall recovery, muscular stress, fatigue, and ACWR), 2 (for mood and sleep quality), and 1 (for HRV).

#### 2.3.3 Training and Match Load

The player session rating of perceived exertion (PSRPE) was extracted from the ACWR subscale of the LRS. It was calculated as the product of the subjective rating of perceived exertion (0–10) and the duration of daily training sessions in minutes (Haddad et al. 2017). Coaches recorded their intended player load using the same method (trainer session rating of perceived exertion, TSRPE) prior to the respective session but as a generalization for the entire team via a workload planning spreadsheet.

The GPS‐ and accelerometry‐based parameters “total distance covered (TD)” and “total distance above 20 km/h (TD20)” were recorded using the Catapult Vector S7 (Catapult Sports Pty Ltd, Boston, Massachusetts, USA) 10 Hz units (U21 and U18) and the Polar Team Pro 10 Hz (Polar Electro Oy, Kempele, Finland) system (U19). Both systems are valid and reliable, with high inter‐unit reliability (Akyildiz et al. 2021; Akyildiz et al. 2022; Johnston et al. 2012, 2014). For more detailed information about the different data collection measures; see Appendix A, Table A1.

### 2.4 Statistical Analysis

The design and conduct, data analysis, reporting, presentation, and interpretation of the data were assessed using the CHecklist for statistical Assessment of Medical Papers (CHAMP) (Mansournia et al. 2021). All statistical analyses were computed using the statistical software R version 4.2.3 with the RStudio environment (v3.0.386). The demographic data of the sample were described using means, standard deviations, and frequencies. For the primary analyses, the functionalities of the package lme4 (v1.1‐32) (Bates et al. 2015) were used. To examine the direction and strength of the relationship between the LRS and the abovementioned measures of training and match load, various linear mixed‐effects models (LMM) were fitted via restricted maximum likelihood (REML). The model assumptions of linearity, homogeneity of variances/homoscedasticity, and normal distribution of residuals apply (Bates et al. 2015). Random intercepts were defined for each player to account for the repeated within‐subject measurements (Fisher et al. 2018; Molenaar and Campbell 2009; Neumann et al. 2021), and the demographic control variables height, body mass, and sex were included in the models. The significance level was set to α = 0.05. For the interpretation of the correlation of fixed effects (*ρ* = Cov (*X*_1_, *X*_2_)/σ_1_σ_2_), the following thresholds were used (Portney 2020): ≤ 0.25, little or no relationship; 0.25–0.50, low to fair; 0.50–0.75, moderate to good; ≥ 0.75, strong relationship. Furthermore, the variance explained by the random effects was calculated using Nakagawa's marginal and conditional *R* ^2^ for mixed models (Nakagawa et al. 2017). Missing data are assumed to be missing at random (MAR) (Rubin 1976), which the LMM handles via listwise deletion and, therefore, using complete record analysis. The results will be unbiased if the missingness is MAR or MCAR but will be less precise than if we had observed all the data (Carpenter and Smuk 2021).

## 3 Results

### 3.1 Participants

The initial sample consisted of 78 players. Two players with long‐term illnesses or injuries were excluded based on the defined eligibility criteria. Five additional players were suspended for noncompliance. The final sample included 71 players (32.4% female) with a mean age of 17.9 years (SD = 1.2). See Table 1 for the demographic characteristics of the final sample.

**TABLE 1:** Demographic characteristics of the sample.

Variable	Overall, *n* = 71^a^	Female, *n* = 23^a^	Male, *n* = 48^a^
Team			
U18	28	0	28
U19	23	23	0
U21	20	0	20
Playing position			
Defender	31 (44%)	12 (52%)	19 (40%)
Midfielder	18 (25%)	3 (13%)	15 (31%)
Forward	14 (20%)	6 (26%)	8 (17%)
Goalkeeper	8 (11%)	2 (9%)	6 (12%)
Age	17.9 (1.2)	17.7 (0.8)	18.0 (1.4)
Height (cm)	176.7 (9.9)	165.8 (5.0)	181.7 (7.1)
Body mass (kg)	68.3 (10.2)	58.1 (5.8)	73.5 (7.7)

^a^ Mean (SD) or frequency (%).

### 3.2 Descriptive Statistics of the Measures

Descriptive statistics and missing data points from all used measures are reported in Table 2. Missings of LRS and PSRPE values were high (49.2%/78.27%) in the U18 and U21 teams (male). Nevertheless, for the dependent variable LRS, 1268 daily values were recorded, representing an average of 26 values from each of the 48 male players.

**TABLE 2:** Descriptive summary of the measures.

Sex	Variable	*n*	Mean (SD)	Range	Skew	Kurtosis	*n* missings (%)
Female	Load and recovery score^a^	744	89.45 (10.21)	54–114	−0.38	−0.25	61 (7.58)
	Player‐SRPE^b^	424	464.34 (188.39)	10–1050	0.47	0.22	36 (7.83)
	Trainer‐SRPE^b^	460	568.25 (212.35)	350–900	0.59	−1.33	0 (0)
	Total distance covered (km)^c^	102	5.81 (2.75)	0.66–12.24	0	−0.73	36 (26.09)
	Total distance > 20 km/h (m)^c^	102	195.12 (172.37)	0–725	0.91	0.25	36 (26.09)
Male	Load and recovery score^a^	1268	91.11 (11.45)	52.5–119	−0.25	−0.04	1228 (49.2)
	Player‐SRPE^b^	392	438.35 (197.97)	10–1200	0.53	0.4	1412 (78.27)
	Trainer‐SRPE^b^	1804	434.62 (192.13)	120–805	0.75	−0.52	0 (0)
	Total distance covered (km)^c^	822	5.39 (2.09)	0.07–11.95	0.73	0.43	466 (36.18)
	Total distance > 20 km/h (m)^c^	822	247.27 (248.88)	0–1223.84	1.23	0.89	466 (36.18)

^a^ Possible data points: U19 (female) 35 days × number of players, U18/U21 (male) 52 days × number of players.

^b^ Session rating of perceived exertion; possible data points from match and training days only: U19 20 days × number of players, U18/U21 38/37 days × number of players.

^c^ Possible data points: U19 6 days × number of players, U18/U21 26/28 days × number of players.

### 3.3 Inferential Analyses

**FIGURE 1:**
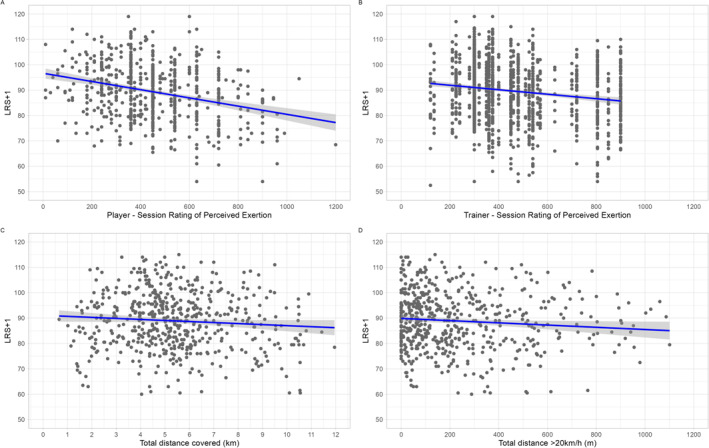
Linear regression models with 95% confidence intervals for the influence of the (A) PSRPE, (B) TSRPE, (C) TD (km), and (D) TD20 (m) on the following day's load and recovery score (LRS + 1).

The analysis of the linear mixed‐effects model via restricted maximum likelihood for the influence of the PSRPE on the LRS of the following day (LRS + 1) when controlled for the repeated within‐subject measurements showed significant fixed effects (−0.013, 95% CI [−0.017, −0.010], *p* < 0.001) (Figure 1A). Specifically, for every one‐unit increase in PSRPE, the LRS + 1 decreased by 0.013 points (95% CI [−0.017, −0.010]). This translates to a drop of about 5.9 points on the LRS + 1 when PSRPE increases from zero to its mean value of 452, highlighting a practically relevant relationship between higher perceived exertion and reduced recovery status. The correlation of fixed effects was −0.615, indicating a moderate to strong inverse relationship, with the residual factor explaining 44% of the variance (marginal *R* ^2^ = 0.053, conditional *R* ^2^ = 0.497). This model included 666 data points from 65 players. The same model approach was used to see how the LRS + 1 and the TSRPE are related. Here, each unit increase was associated with a 0.008‐point drop in the LRS + 1 (95% CI [−0.011, −0.006], *p* < 0.001) (Figure 1B). This equates to a decrease of about 3.7 points for an increase from zero to the mean TSRPE value of 462. Again, the correlation of fixed effects was moderate to good (−0.514), and the residual variance explained was comparable (marginal *R* ^2^ = 0.022, conditional *R* ^2^ = 0.466; *N* = 1350 observations from 71 players).

TD showed a significant fixed effect (−0.668, 95% CI [−0.979, −0.355], *p* < 0.001) (Figure 1C) on the LRS + 1 when controlled for the repeated within‐subject measurements. The correlation of fixed effects was −0.602, again showing a moderate to good inverse relationship. With an increase from 0 to 5.435 km (mean value) of the TD, the LRS + 1 would drop by 3.6 points. The variance explained by the residual factor (ID) is 0.513 (marginal *R* ^2^ = 0.017, conditional *R* ^2^ = 0.530). The model included 551 data points from 58 players. Furthermore, TD20 showed a significant fixed effect (−0.009, 95% CI [−0.012, −0.006], *p* < 0.001) (Figure 1D) on the LRS + 1 when controlled for the repeated within‐subject measurements. With an increase from 0 to 242 m (mean value) of the TD20, the LRS + 1 would drop by 2.2 points. The correlation of fixed effects was weaker (−0.271), suggesting a low to fair association. The variance explained by the residual factor (ID) is 0.526 (marginal *R* ^2^ = 0.033, conditional *R* ^2^ = 0.559). The model included 551 data points from 58 players. Taken together, these results demonstrate that both subjective (PSRPE, TSRPE) and objective (TD, TD20) load indicators have practically relevant, negative relationships with next‐day recovery status. As a sensitivity analysis, different subject characteristics (e.g., sex, height, and body mass) were included in the abovementioned models. The addition of control variables did not significantly influence the direction or magnitude of the model's effects (Table A2 in Appendix B for results of sensitivity analyses).

**FIGURE 2:**
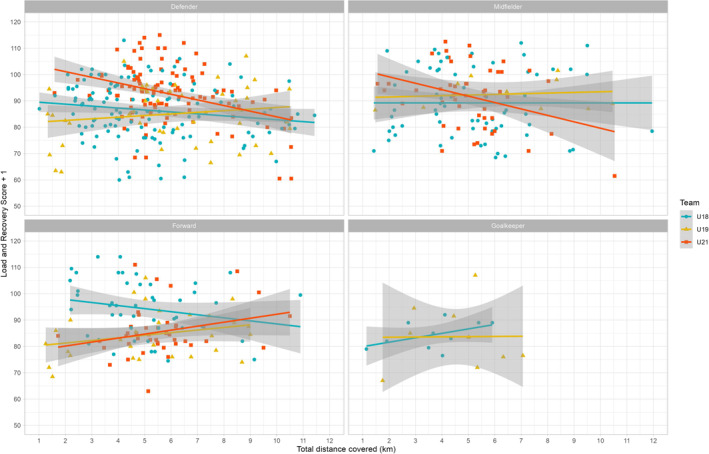
Post‐hoc linear regression models with 95% confidence intervals for the influence of the load variable “total distance covered” in km on the following day's load and recovery score (LRS + 1), presented separately for the respective playing positions and teams; the U18 and U21 teams play in male divisions, and the U19 team plays in a female division.

Post‐hoc analyses were conducted to explore whether playing position or sex influenced the relationship between load parameters and the following day's LRS (LRS + 1). In the U19 team (female), both TD (Figure 2) and TD20 showed nonsignificant but positive fixed effects on LRS + 1. Similarly, for forwards in the U21 male team, TD and TD20 showed small positive trends (Figure 2). Comparing playing positions more broadly, defensive players tended to show higher (more negative) fixed effects of all load parameters on LRS + 1 compared to forwards, midfielders, and goalkeepers (exemplary illustration for TD in Figure 2).

## 4 Discussion

This study aimed to explore whether the developed LRS is associated with various training and match load variables in Swiss top‐level youth soccer players and whether it could, therefore, serve as a suitable tool for assessing the constructs of load and recovery. Our results indicate that the LRS shows significant negative associations with training and match load measures, such as the PSRPE, TSRPE, TD, and TD20, when controlled for repeated within‐subject measurements. This means that higher training and match loads are consistently reflected in lower LRS scores on the following day, highlighting the scale's capacity to capture the fatigue and recovery state of players. This is an important finding because it demonstrates that the LRS is sensitive to day‐to‐day fluctuations in physical and perceived exertion, supporting its use as a practical monitoring tool. By providing timely feedback on whether athletes are adequately recovering from training demands, the LRS can help coaches adjust individual or team training loads to optimize performance and reduce the risk of overtraining or injury. The PSRPE and the TSRPE have been established as a noninvasive, accessible, valid, and reliable method to monitor training load (Haddad et al. 2017; Inoue et al. 2022; Paul et al. 2021). The present results demonstrate that the PSRPE and TSRPE, which are widely used subjective ratings of training load, are reflected in the LRS. The strongest negative association was observed between LRS + 1 and PSRPE, indicating that higher perceived exertion by players was associated with lower self‐reported recovery and readiness the following day. This aligns with previous research highlighting the utility of subjective measures in monitoring internal load and their sensitivity to fatigue and recovery dynamics (Haddad et al. 2017; Saw et al. 2016). Similarly, TSRPE demonstrated a significant inverse association with LRS + 1, albeit slightly weaker than the PSRPE. This is consistent with earlier findings suggesting that although coach‐rated exertion correlates with player perceptions, individual internal responses are more accurately captured through self‐reports (Brink et al. 2010; Inoue et al. 2022).

The objective external load indicators, TD and TD20, were also significantly negatively associated with LRS + 1. Although these associations were generally weaker than those found for subjective measures, they provide complementary evidence supporting the LRS's responsiveness to training and match load. The significant inverse association between TD and LRS + 1 indicates that a higher total distance covered during a session correlates with a lower recovery status the following day. Although TD is likely the most commonly monitored variable in elite soccer, it has been shown not to be associated with changes in any postgame fatigue‐related markers (Hader et al. 2019). However, TD20 has been demonstrated to be a sensitive monitoring variable, characterizing biochemical and neuromuscular responses during the initial 24‐h postmatch recovery period (Hader et al. 2019). Notably, TD20 had the weakest fixed‐effect correlation with LRS + 1. This may reflect the multifactorial nature of recovery, which is influenced not only by high‐intensity bouts but also by overall session load, individual fitness, and psychological factors (Bourdon et al. 2017). The lower correlation might also be attributed to greater interindividual variability in high‐speed running tolerance or positional differences in match and training demands (Dellal et al. 2010).

Sensitivity analyses confirmed that adding covariates such as sex, height, and body mass did not materially alter the strength or direction of the observed effects, indicating the robustness of the core models. However, post‐hoc analyses uncovered nuanced patterns worth noting. Notably, in female players (U19 team) and male forwards (U21), the associations between load metrics and LRS + 1 were not statistically significant and, in some cases, even trended positively. This suggests potential variability in how recovery is perceived and experienced across different subgroups, possibly reflecting physiological, psychological, or contextual differences (e.g., match exposure, tactical roles, hormonal influences).

Furthermore, although not statistically significant, defensive players appeared to experience greater decrements in recovery from similar training loads compared to other positions. This could reflect the higher physical demands typically associated with defensive roles, such as repeated high‐intensity efforts and increased involvement in transitions and duels (Dellal et al. 2010).

**FIGURE A1:**
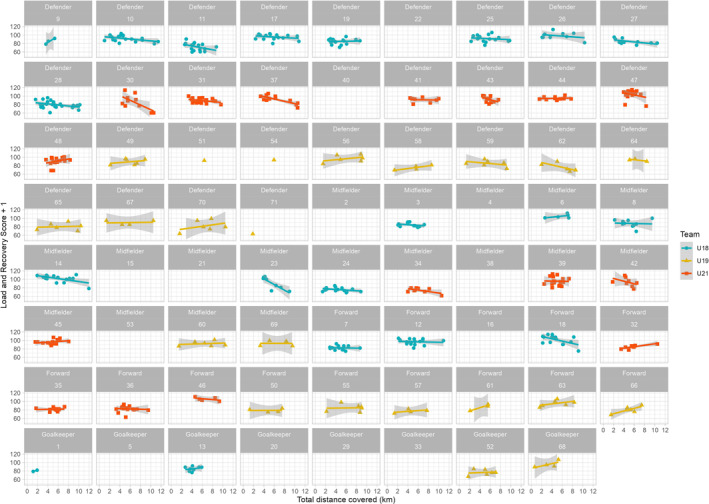
Linear regression models with 95% confidence intervals for the influence of the load variable “total distance covered” in km on the following day's load and recovery score (LRS), presented separately for each ID (*n* = 71), the respective playing positions, and teams; the U18 and U21 teams play in male divisions, and the U19 team plays in a female division.

Despite the significant associations observed in this study, it is important to note that the variance explained by the fixed effects of load variables in our models was relatively small compared to the residual variance explained by individual differences. Consistent with current research (Hader et al. 2019; Neumann et al. 2021; Wiig et al. 2019), our findings show that load variables generally influence recovery parameters, although these effects can vary among certain groups (e.g., playing position or sex; see Figure 2) and specifically among individuals (Figure A1 in Appendix C for individual scores). Therefore, athlete monitoring and subsequent adjustments aimed at performance optimization and injury risk reduction should be based on individual‐level data (Bourdon et al. 2017; Neumann et al. 2021). In practical terms, although a single fixed effect in a linear model can provide a summary of the relationship between load and recovery, it is important to monitor individual variables continuously. The LRS should be viewed as part of a broader athlete monitoring strategy that includes both subjective and objective measures, ensuring that recovery assessments are accurate and tailored to each athlete's needs.

### 4.1 Practical Applications

By providing individualized and multifactorial insights into training load and recovery, the LRS offers coaches a practical tool to better understand how players respond to daily training and match demands. Its ability to detect fluctuations in an athlete's readiness can help coaches make informed adjustments to training on an individual basis, thereby reducing the risk of injuries, nonfunctional overreaching, and overtraining. Unlike traditional self‐report systems, which typically cover a few subjective factors, the LRS integrates multiple subjective subscales with objective information, aiming to provide a more comprehensive picture of player status. Because the LRS reflects both subjective and objective load information, it can complement existing monitoring routines and support day‐to‐day decision‐making, ultimately contributing to more effective performance management throughout the season. However, as this is the first study to investigate the LRS, clear thresholds for interpretation and concrete recommendations cannot yet be defined. It still requires a coach to interpret trends in the score in the context of other relevant information and make informed, experience‐based adjustments.

### 4.2 Limitations

There are several limitations to consider when attempting to generalize the findings of this study. First, the results derived from this convenience sample of high‐level athletes may not be directly applicable to other populations. This is important because elite players often have distinct physical capacities, as well as greater experience with self‐monitoring and reporting, which may influence how they perceive and report their load and recovery status. Additionally, the sample was not gender‐balanced (32.4% female), which could limit the generalizability of the findings, as sex‐specific physiological and perceptual differences may influence how players experience and report load and recovery. Because of the lack of comparable prior studies, a formal sample size analysis could not be conducted. However, our sample size aligns with those of similar observational studies in elite athlete populations (Halson 2014), supporting its adequacy for initial exploratory analysis in this context. Future studies should therefore investigate the applicability and validity of the LRS in broader, more gender‐balanced samples to confirm its wider relevance.

Second, the lack of a gold standard for assessing training load in team sports may have resulted in the use of a less optimal predictor in the linear mixed models, which could have introduced bias and affected the generalizability of the results. Although we included both objective and subjective parameters, future studies should incorporate additional parameters, particularly those quantifying internal training load.

Third, for a definitive and conclusive statement on the construct validity of the LRS, an exploratory or confirmatory factor analysis should be carried out to determine the number of constructs (presumably two, load and recovery), which should then be validated individually. Moreover, the instrument can only be considered valid for measuring a particular construct if two conditions are met: First, the construct must exist, and second, changes in the construct must lead to corresponding changes in the measurement outcomes (de Vet et al. 2011).

Fourth, we only assessed the impact of load and recovery measures on the LRS + 1. However, because research indicates that, for example, muscle damage markers and physical and well‐being status take much longer to return to baseline (Silva et al. 2018), it would be recommended to investigate the residual impact of different load and recovery measures on the LRS after 48 h, 72 h, and even 96 h. Fifth, we did not control for potential contextual factors such as environmental conditions or other situational stressors. These variables could have influenced both training load and recovery status, potentially confounding the observed relationships. Additionally, the limited availability of GPS data for the U19 team may have reduced the precision of subgroup comparisons, particularly for analyses involving TD and TD20. Finally, variable compliance levels—particularly lower compliance observed in the male teams—may have resulted in an overrepresentation of data from more compliant players in the analyses. This could potentially bias the findings toward the responses of those who consistently provided data. Although this limitation may not substantially affect the internal validity of our main analysis, it does raise concerns regarding the generalizability of results to players with lower compliance or less consistent monitoring. Practical barriers to compliance included players' busy schedules, competing priorities such as school or work, and occasional technical issues with the web application. To improve adherence in future studies, strategies such as regular reminders via notifications or messages, providing incentives for consistent participation, simplifying the data entry process, and involving coaching staff to encourage compliance could be implemented.

## 5 Conclusion

This study provides initial evidence that the LRS is a promising tool for tracking daily fluctuations in players' recovery status in relation to training and match loads. By showing consistent associations with subjective and objective load measures, the LRS demonstrates potential to complement existing monitoring practices in elite team sports. However, as the variance explained by the load variables was modest compared to individual differences, the LRS should be interpreted within a comprehensive, athlete‐centered monitoring approach. Future research should validate its use in other age groups, competitive levels, and sports contexts; explore its integration with injury surveillance data; and examine how it can be practically embedded in daily coaching routines to better support player health and performance.

## Ethics Statement

This study was approved on the fifth of July, 2022, by the Ethics Committee of the Faculty of Humanities at the University of Bern (Review No. 2022‐06‐00008).

## Consent

All participants gave informed consent. For participants under the age of 18, informed consent from their legal guardian was obtained.

## Conflicts of Interest

The authors declare no conflicts of interest.

## Data Availability

The data supporting this study's findings are available upon request from the corresponding author. The same applies to the code for the statistical analyses.

## Appendix A Measures of Data Collection

**TABLE A1:** Measures of data collection.

Outcome	Measurement method
Demographic data	Provided by team coaches, collected by questionnaire
	Team
	Playing position
	Age
	Height (cm)
	Body mass (kg)
	Current occupation
Load and recovery score (LRS)	Web application
	Physical capability (SRSS)
	General state of regeneration (SRSS)
	Muscular stress (SRSS)
	Fatigue (POMS‐F/BRUMS‐F)
	Mood (VAMS)
	Sleep quality (PSQI)
	HRV (Polar H10, Elite HRV)^a^
	ACWR (player—SRPE)
Training and match load	Session rating of perceived exertion (SRPE)
	Player rating
	Trainer prediction
	GPS tracking and accelerometry (Catapult Vector S7/Polar Team Pro)
	Total distance covered (km)
	Total distance > 20 km/h (m)

^a^ In RMSSD (root mean square of successive differences between normal heartbeats).

Abbreviations: ACWR, acute chronic workload ratio; BRUMS‐F, Brunel Mood Scale–Fatigue; HRV, heart rate variability; POMS‐F, Profile of Mood States–Fatigue; PSQI, Pittsburgh Sleep Quality Index; SRSS, Short Recovery and Stress Scale; VAMS, Visual Analog Mood Scales.

## Appendix B Results of Sensitivity Analyses

**TABLE A2:** Results of sensitivity analyses (linear mixed‐effect models fitted via REML).

Dependent variable	Independent variable	Random effects terms	Fixed effects (95% CI)	Data points	Subjects (*n*)
Load and recovery score	Player—SRPE^a^	ID, sex, age, body mass	−0.013 (−0.016, −0.009)*	622	58
Load and recovery score	Trainer—SRPE^a^	ID, sex, age, body mass	−0.008 (−0.011, −0.006)*	1237	64
Load and recovery score	Total distance covered (km)	ID, sex, age, body mass	−0.543 (−0.858, −0.232)*	506	53
Load and recovery score	Total distance > 20 km/h (m)	ID, sex, age, body mass	−0.009 (−0.012, −0.006)*	506	53

^a^ Session rating of perceived exertion.

^*^ *p* < 0.001.
